# An update on trismus: etiology, diagnosis and treatment

**DOI:** 10.3389/fneur.2026.1758417

**Published:** 2026-03-24

**Authors:** Marco Falletti, Martina Cuccarelli, Enrico Evangelisti, Alessandro Zampogna, Martina Patera, Andrea Truini, Umberto Romeo, Antonio Suppa

**Affiliations:** 1 Department of Human Neurosciences, Sapienza University of Rome, Rome, Italy; 2 Department of Anesthesia and Intensive Care Unit, Campus Bio-Medico University Hospital Foundation, Rome, Italy; 3 IRCCS Neuromed Institute, Pozzilli, Italy; 4 Department of Oral and Maxillofacial Sciences, Sapienza University of Rome, Rome, Italy

**Keywords:** botulin toxin, lockjaw, malignant syndrome, tetanus, trismus

## Abstract

Trismus, or “lockjaw,” is a condition characterised by limited mouth opening due to masticatory muscle dysfunction. Its multifactorial etiology includes neurological and non-neurological causes, but current clinical guidance is fragmented. This narrative review synthesises current evidence on trismus pathophysiology, diagnosis, and treatment. We propose a novel etiological classification distinguishing neurological from non-neurological causes and introduce a stepwise diagnostic and management algorithm. The algorithm prioritises red flag conditions, guides differential diagnosis, and supports treatment decisions in both outpatient and hospital settings. By providing a structured framework, this review aims to facilitate early recognition of the conditions, inform targeted therapeutic strategies, and improve patient outcomes.

## Introduction

Trismus, commonly known as “lockjaw,” is a clinical condition characterised by a persistent, involuntary restriction in the ability to open the mouth due to tonic contraction or spasm of the masticatory muscles (1). The prevalence of trismus remains uncertain, as it varies depending on the underlying patient population and specific etiologies (2, 3). It is often underestimated due to the lack of standardised screening protocols and the challenges in distinguishing trismus from other causes of limited mouth opening, particularly in sedated or critically ill patients (1, 4). Diagnosis is primarily based on clinical assessment and physical examination, while instrumental investigations can aid in identifying the underlying etiology (1, 4). A more comprehensive delineation of the underlying etiological mechanisms of trismus would bridge gaps between pathophysiological insights and current therapeutic management. Also, the development of a structured and evidence-based clinical management algorithm would optimise the clinical management of trismus and improve patient outcomes. Hence, a comprehensive review of the clinical causes of trismus would provide a unified framework for improving its clinical management.

In the first section of this narrative review, we examined the physiology of mastication, with a particular focus on the neural mechanisms underlying its control. Then, after a critical review of the currently available scientific literature on trismus, we proposed a novel etiological classification aimed at systematically categorising the primary causes of trismus. The classification distinguishes between *neurological* and *non-neurological* factors, each of which is further subdivided into *structural* and *non-structural* causes of trismus. Finally, building upon this classification, we propose a structured algorithm to guide the diagnostic process and the clinical management of trismus, encompassing both clinical and surgical approaches, including treatment considerations in a critical care setting.

## The masticatory function

### Anatomy and physiology of the masticatory function

Masticatory function is an elaborate physiological process that allows the mechanical reduction of food into small particles to be swallowed and enzymatically digested. It involves skeletal, articular, dental, muscular, glandular, and receptor structures that together compose the stomatognathic system. The mandible and maxilla represent the bony bases that support this function (5). The mandible is the only movable bone of the facial skeleton, articulating bilaterally with the temporal bone through the temporomandibular joints (TMJs) (5–7). In comparison, the maxilla is a stationary bone. It provides a stable support for the upper dental arch, in close anatomical relationship with craniofacial structures such as the zygomatic and palatine bones. The relationship between mandible and maxilla, mediated by occlusal contacts, is essential for stability and efficiency of mastication (8). Teeth, symmetrically aligned in the arches, are classified as incisors, canines, premolars, and molars. Incisors, with their sharp edges, are suited for cutting; canines, with robust cusps, pierce food and guide mandibular movements; premolars and molars, with broad and cuspidated occlusal surfaces, are specialized for grinding and crushing the food bolus (5, 8). Dental support is provided by the periodontal ligament, which connects the root to the alveolar bone and acts as a shock absorber during occlusal loading. Within it, several mechanoreceptors are distributed, contributing to the perception of occlusal pressure and the protection of dental and periodontal tissues (8, 9). TMJs, thanks to their condyle–disc structure, allow both rotational and translational movements, allowing mandibular opening, closing, lateral excursions, and protrusion. The fibrocartilaginous articular disc promotes congruence between the osseous surfaces and the homogeneous distribution of mechanical stresses (6, 7). Masticatory muscles include the masseter, temporalis, medial and lateral pterygoid. These muscles act synergistically to generate force and guide mandibular movements. The masseter and temporalis are primarily responsible for jaw elevation, whereas the Pterygoid muscles enable more complex lateral and protrusive movements. Accessory muscles, such as the buccinator and suprahyoid group, assist in bolus positioning and mandibular stabilization (5, 6, 8). Saliva and tongue function are two additional essential components in this process. Saliva, secreted by the major and minor salivary glands, lubricates the oral cavity, softens ingested material, and begins enzymatic digestion through amylase and lipase. It also facilitates bolus cohesion, allowing food particles to be bound into a mass suitable for swallowing. Tongue plays a dual role: it continuously repositions food between occlusal surfaces during chewing and shapes the bolus in cooperation with saliva. Intrinsic and extrinsic tongue muscles ensure both slight adjustments and major movements, making the tongue indispensable not only for mastication but also for swallowing and phonation (5). Mastication remains adaptable owing to the integration of anatomical and receptor components, even though it is largely automatic. In this context, periodontal ligament receptors, together with sensory input from the oral mucosa and tongue, play an essential role in modulating muscular activity and preserving the integrity of the structures involved (8, 9).

### The neural control of the masticatory function

Mastication (or chewing) is a highly coordinated, rhythmic motor behaviour involving complex interactions between central pattern generation, cortical modulation, and peripheral sensory feedback (10). The basic rhythm of chewing seems to be generated by a brainstem central pattern generator, with key rhythmogenic elements located in the dorsal division of the main sensory nucleus of the trigeminal nerve (11). Neurons in this region have intrinsic oscillatory properties and have been shown to drive rhythmic activation of the motor nucleus of the trigeminal nerve, as demonstrated by both electrophysiological recordings and calcium imaging (12). The trigeminal motor nucleus, located in the pons, contains jaw-opening and jaw-closing motoneurons, segregated into a ventromedial cluster and a dorsolateral cluster, respectively, with the latter being significantly larger and comprising mostly motoneurons for masseter and temporalis muscles (13). These motoneurons receive descending input from both the rhythmogenic network and higher-order cortical centres, as well as excitatory and inhibitory interneurons within the brainstem. Cortical structures also play a critical role in initiating and modulating masticatory behaviour. The cortical masticatory area, located in humans within or adjacent to the primary motor cortex, is the only cortical region whose inactivation significantly impairs chewing, although other areas such as the primary somatosensory cortex, premotor cortex, supplementary motor area, insular cortex, anterior cingulate cortex, and amygdala contribute to the modulation of orofacial motor output (14). These regions send direct and indirect projections to brainstem premotor areas, including the peritrigeminal region, the parvocellular reticular formation, the intermediate reticular formation, and the spinal divisions of the trigeminal sensory nucleus, namely the subnucleus oralis and the subnucleus interpolaris. Notably, parallel descending pathways from distinct but adjacent cortical territories innervate partially overlapping populations of premotor neurons, allowing for flexible control of jaw and related limb movements (15). Peripheral sensory feedback is indispensable for the fine-tuning of mastication. Periodontal mechanoreceptors, embedded within the ligament connecting the teeth to the alveolar bone, provide detailed information about food contact and intraoral mechanical forces (16). Periodontal mechanoreceptors are subclassified into fast-adapting receptors, primarily located in the molars and active during the initial phase of jaw closure, and slow-adapting receptors, found in the incisors and active throughout the entire closing phase (17). Inactivation of periodontal mechanoreceptors results in significant disruption of the masticatory sequence, including impairments in bite force regulation and fine oromotor control (18). These receptors, along with muscle spindles (particularly enriched in the deep layers of the masseter muscle), contribute to orofacial proprioception. While spindles are absent in jaw-opening muscles, they are prevalent in jaw-closing muscles and convey afferent signals via monosynaptic or disynaptic pathways to trigeminal motoneurons and hypoglossal motor nucleus, as well as to integrative centres such as the cerebellum, nucleus ambiguous, principal sensory nucleus of the trigeminal nerve, spinal trigeminal nucleus, parvocellular reticular formation, and intermediate reticular formation (19). Rhythmic jaw movements can be evoked by stimulation of multiple cortical and subcortical sites, but only the cortical masticatory area is required for the maintenance of chewing under physiological conditions. Coordination of mastication with other oral movements, such as swallowing and speech, requires complex timing and integration within brainstem and forebrain circuits (15). Functional segregation within brainstem nuclei supports this flexibility: the subnucleus oralis is more associated with jaw motor control, while the subnucleus interpolarisoral is more engaged in forelimb coordination. Many studies have mapped premotor interneurons projecting to jaw - and tongue-innervating motoneurons. Among these, those potentially involved in rhythm and/or pattern generation of masticatory movements, likely to fire rhythmically during rhythmic jaw movements, are located in the peritrigeminal region, parvocellular reticular formation, intermediate reticular formation, and the sensory complex (20). The peritrigeminal region, a ring-shaped region surrounding the trigeminal motor nucleus, has its largest concentration of premotoneurons in the dorsal supratrigeminal region. Tracing studies show that this region receives inputs from the masseter, inferior alveolar, and infraorbital nerves, along with bilateral corticofugal projections from primary and secondary somatosensory areas, and the granular insular cortex. It projects to several areas, including peritrigeminal region subdivisions, the cerebellum, the ventral posteromedial thalamic nucleus, and the trigeminal motor nucleus. Evidence suggests supratrigeminal region is more likely involved in pattern generation than in rhythm generation (15). Overall, a comprehensive understanding of the functional neuroanatomy underlying mastication constitutes an essential prerequisite for a rigorous interpretation of the pathophysiological mechanisms responsible for trismus. Such knowledge not only facilitates the identification of the neural substrates involved but also provides the conceptual framework necessary to develop targeted diagnostic approaches and more effective therapeutic strategies.

## Literature research strategy and criteria

This narrative review was conducted following the SANRA (Scale for the Assessment of Narrative Review Articles) recommendations (see Supplementary material). Literature research of studies investigating trismus was performed using the following databases: MEDLINE, Scopus, PubMed, Web of Science, EMBASE and the Cochrane Library. The search terms included “trismus,” “jaw dysfunction” and “treatment of trismus”. Specific terms such as “botulinum toxin type A” and “BoNT-A” for trismus were also included. Boolean operators (AND, OR) were used to refine the search results. Additionally, search terms related to tetanus, such as “tetanus trismus” and “tetanus lockjaw,” were included. For this narrative review, studies were included if they were published in peer-reviewed journals and provided clinically relevant data, case reports, case series, or expert reviews addressing the etiology, pathophysiology, diagnosis, or management of trismus. We considered studies reporting interventions such as pharmacological therapy, botulinum toxin type A (BoNT-A), physical therapy, surgical procedures, or rehabilitation strategies. Both neurological and non-neurological causes, including structural and non-structural factors, were considered. Studies were excluded if they were not published in English, focused solely on animal models or *in vitro* experiments without translational relevance, or lacked direct clinical applicability. Editorials, letters, conference abstracts, non-peer-reviewed publications, and anecdotal reports without sufficient clinical or scientific detail were also excluded. As this is a narrative, non-systematic review, these criteria were used as a guide for literature selection while allowing flexibility to incorporate seminal, highly cited, or otherwise clinically significant references, even when they did not strictly meet all inclusion criteria. The reference lists of retrieved articles were also manually searched for additional studies. Data were synthesised into thematic categories to provide a coherent narrative analysis. Based on this analysis, we here propose a refined etiological classification of trismus, distinguishing between *neurological* and *non-neurological* factors. Each category is further stratified into *structural* and *non-structural* causes. Neurological etiologies are additionally differentiated according to the involvement of either the central nervous system (CNS) or the peripheral nervous system (PNS).

## Etiology and pathophysiology

According to the etiology and pathophysiology, trismus may be categorised as either *neurological* or *non-neurological*, *structural* or *non-structural*, both ultimately resulting from dysfunction of the masticatory system. A new etiological classification of trismus based on this classification is summarised in Table 1.

**Table 1:** Differential diagnosis of trismus.

	Neurological causes		Non-neurological causes
	CNS	PNS	
Structural	Traumatic brain injury (TBI)	Trigeminal nerve lesions	TMJ disorders (dysfunction or ankylosis)
	Diffuse axonal injury (DAI)		Tumours of the oral cavity or oropharynx, or of the masticator space
	Brain tumours		Trauma
	Stroke (ischemic or haemorrhagic)		Orofacial infections (peritonsillar abscess, dental abscess, osteomyelitis)
	Multiple sclerosis		Fractures of the mandible or maxilla
	CNS infections: meningitis and encephalitis		Post-surgical oedema or hematoma following dental or maxillofacial procedures
			Surgical complications
			Soft tissue injuries causing hematoma or oedema.
			Radiation therapy
			Fibrosis due to prolonged immobility
			Burns
			Complication of dental extraction (impacted third molar)
			Rheumatoid arthritis
			Scleroderma
			Temporal arteritis
			Congenital malformation: trismus pseudo-camptodactyly syndrome, arthrogryposis multiplex congenital
Non-structural	Status epilepticus	Tetanus	Heavy metal poisoning (lead or mercury)
	Parkinson’s disease and atypical parkinsonian syndromes	Trigeminal neuralgia	Hypocalcaemia, hypomagnesemia
	Storage disease (i.e., Wilson’s disease)		Anesthetics medications (succinylcholine, halothane, etomidate)
	Medication adverse effects or poisoning (phenothiazines, metoclopramide, etc.)		Muscle contractures
	Functional disorder		Myofascial pain syndrome
	Malignant syndrome		Malignant hyperthermia
	Status dystonicus and OMD		
	Hemimasticatory spasm		
	Uremic encephalopathy		

### Neurological causes: structural damage to the central or peripheral nervous system

Trismus can arise from various structural lesions to the CNS, particularly when motor pathways involved in the masticatory function are disrupted. In diffuse axonal injury (DAI), a subtype of traumatic brain injury (TBI), widespread shearing of white matter tracts compromises both voluntary and reflex-based regulation of orofacial movements. This dysregulation often manifests as increased tone of the masticatory muscles, thereby contributing to the development of trismus (21). Similarly, post-stroke trismus is most frequently associated with lesions affecting the corticobulbar tract. Damage along this pathway leads to disinhibition of brainstem motor nuclei, resulting in hyperactivity and sustained contraction of the jaw-closing muscles (22). Furthermore, lesions of the brainstem, whether caused by demyelinating, neoplastic, inflammatory, or traumatic disorders, can interfere with the descending inhibitory control that normally regulates masticatory motor output. Loss of this inhibitory modulation favours pathological increase of tone in masticatory muscles, producing persistent and often severe jaw closure (23, 24). Collectively, these findings highlight the pivotal role of CNS integrity in maintaining the physiological motor control of mastication and underscore the need for early recognition of central neurological causes when evaluating patients with trismus.

Besides structural damage of the CNS, specific lesions of the PNS can induce trismus through neuroinflammatory and hyperexcitability mechanisms (25). Trauma or iatrogenic injury to the trigeminal mandibular division (V3) during dental surgery, such as nerve blocks or extractions, may disrupt axonal integrity and trigger inflammatory cascades, releasing mediators like bradykinin and adenosine triphosphate (26). These mediators promote mechanisms of *peripheral sensitisation* by modulating ion channel activity, particularly increasing T-type Ca^2+^ and hyperpolarisation-activated cyclic nucleotide-gated currents in trigeminal ganglion neurons, resulting in abnormal neuronal excitability and spontaneous ectopic discharges. This neuronal activity propagates along motor fibers, causing sustained contraction of the masseter, temporalis, and pterygoid muscles. Furthermore, direct nerve compression or post-traumatic fibrosis can impair normal inhibitory signalling between antagonistic jaw muscles, exacerbating trismus. While central lesions primarily affect supranuclear pathways, structural damage to the PNS underscores how localised neuropathic changes, including ion channel dysregulation, ectopic firing, and inflammatory fibrosis, may directly generate and sustain trismus. In conclusion, a thorough clinical evaluation, complemented by instrumental investigations using neuroimaging (i.e., MRI and CT scans) and neurophysiological techniques that target the trigeminal nerve (i.e., trigeminal reflexes), is indispensable for accurately delineating the pathophysiological mechanisms underlying trismus, enhancing the diagnostic precision and guiding individualised therapeutic strategies.

### Non-structural damage to the central or peripheral nervous system

Within the CNS, several neurological conditions can contribute to the development of trismus. Under a specific scenario, Parkinson’s disease (PD) and atypical parkinsonian syndromes such as Progressive Supranuclear Palsy (PSP) and Corticobasal Degeneration (CBD) can lead to dysregulation of masticatory muscles, thereby promoting trismus (27, 28). Also, trismus may present as a clinical manifestation of oromandibular dystonia, particularly the jaw-closing subtype, or in severe cases as part of status dystonicus (29–33). Epileptic status, through sustained hyperactivation of cortical and subcortical motor circuits, can also precipitate involuntary contraction of the masticatory muscles, resulting in trismus (34). Another group of disorders that can lead to trismus includes storage diseases, often of genetic origin, such as copper (Wilson’s disease) and iron (Neurodegeneration with brain iron accumulation—NBIA) (35, 36). Moreover, hemimasticatory spasm, a rare focal movement disorder, is characterised by paroxysmal, unilateral contractions or twitches of the jaw-closing muscles, which may lead to episodic or persistent trismus (37). Iatrogenic causes should also be considered, as the side-effects of specific medications affecting central neurotransmission and including phenothiazines, fluoxetine, metoclopramide, and tricyclic antidepressants, which have been reported to induce trismus through central motor side effects (38, 39). Uremic encephalopathy, commonly seen in end-stage renal disease, is associated with movement disorders, including trismus, likely due to the accumulation of neurotoxic metabolites (40). Lastly, trismus may be a symptom of functional disorders, such as functional dystonias (41), or of certain psychiatric conditions, including catatonia (42, 43). Collectively, these findings underscore the diverse CNS-related mechanisms underlying trismus, ranging from neurodegenerative conditions to drug-induced motor disorders.

Regarding non-structural damage to the PNS, tetanus represents one of the most well-recognised causes of trismus (44, 45). The condition is caused by *Clostridium tetani*, a spore-forming bacterium that produces tetanospasmin, a potent neurotoxin. Tetanospasmin binds to presynaptic membranes of inhibitory neurons in the spinal cord and brainstem, blocking the release of gamma-aminobutyric acid and glycine. This disruption of inhibitory neurotransmission leads to generalised hyperexcitability of motorneurons, resulting in sustained, involuntary contractions of skeletal muscles, including the masseter and other jaw-closing muscles (44, 45). Clinically, this manifests as the characteristic “lockjaw” of tetanus, which often precedes systemic manifestations of the disease. The severity and duration of trismus correlate with the extent of toxin-mediated neuronal inhibition, and early recognition is critical for prompt management with antitoxin therapy, supportive care, and muscle relaxants (44, 45). Understanding tetanus-induced trismus highlights the broader role of peripheral neurotoxins in disrupting normal motor control and provides a model for the study of toxin-mediated hypertonia in the PNS (44, 45). Moreover, exposure to heavy metals, particularly lead and mercury, can induce neurotoxicity that disrupts normal motor neuron function, leading to increased muscle tone and spastic contractions, including those of the masticatory muscles (46). Collectively, when structural lesions of the CNS or PNS are excluded, a detailed anamnestic and pharmacological history, combined with neurophysiological investigations (i.e., EEG, EMG and trigeminal reflex), and finally evaluation for botulinum toxin exposure, is essential for the accurate characterisation of trismus. This integrated approach allows for the differentiation between organic (i.e., drug-induced), functional or psychogenic etiologies, and tetanus-induced trismus, thereby guiding appropriate diagnostic and therapeutic strategies.

### Non-neurological causes: structural damage to the masticatory system

Trismus encompasses a broad spectrum of structural and inflammatory conditions that restrict mandibular movement through mechanical obstruction, fibrosis, or joint dysfunction. Congenital malformations, including trismus pseudo-camptodactyly syndrome, arthrogryposis multiplex congenita, involve musculoskeletal anomalies or fibrous contractures that restrict jaw mobility (2, 47).

TMJ disorders represent a major non-neurological cause of trismus in dental and maxillofacial practice. Conditions such as internal derangement, inflammatory joint diseases, degenerative osteoarthritis, and TMJ ankylosis can lead to progressive restriction of mandibular mobility through mechanical obstruction, joint inflammation, pain-related muscle guarding, or bony fusion (47–49). Tumours of the oral cavity, oropharynx, or masticator space may invade or compress the masticatory muscles or TMJ, resulting in restricted jaw opening due to pain, tissue infiltration, or mass effect (50). Trauma to the face can cause direct muscle injury, joint disruption, or bleeding into soft tissues, leading to protective spasm or fibrosis (51). Similarly, odontogenic and orofacial infections represent a common cause of trismus in dental settings. Conditions such as pericoronitis, periapical or periodontal abscesses, deep fascial space infections, peritonsillar abscesses, and osteomyelitis induce localized inflammation, edema, and pain, which may provoke reflex masticatory muscle guarding or direct involvement of masticatory spaces, ultimately restricting mandibular movement and requiring timely dental intervention (52). Mandibular or maxillary fractures may mechanically obstruct mandibular movement or lead to fibrotic healing. Post-surgical trismus is a common complication following dental and maxillofacial procedures. Edema, hematoma formation, and inflammation after interventions such as complicated third molar extractions, orthognathic surgery, or implant placement may compress adjacent structures and restrict mandibular mobility (53). Moreover, surgical trauma to masticatory muscles, prolonged mouth opening, local anesthetic injections, and postoperative fibrosis or excessive scar formation may further contribute to transient or persistent limitation of jaw movement (54). Radiation therapy, particularly in head and neck cancer patients, induces vascular damage and chronic inflammation promote progressive fibrosis and loss of elasticity of the masticatory muscles, TMJ structures, and surrounding connective tissues, leading to reduced mandibular mobility (55). Prolonged immobility of the jaw, whether postoperatively or due to pain, may similarly promote fibrosis and contracture. Thermal injuries, such as facial burns, can cause skin and subcutaneous tissue contracture, mechanically limiting mandibular opening (56). Lastly, autoimmune diseases such as rheumatoid arthritis and scleroderma contribute through synovial inflammation, fibrosis, and soft tissue tightening, respectively, while temporal arteritis may impair vascular supply to the masticatory muscles, leading to ischemia-induced dysfunction (57). Hence, trismus emerges as a complex phenomenon, arising from a wide variety of congenital, traumatic, inflammatory, neoplastic, or iatrogenic conditions that, through different mechanisms, converge in limiting mandibular opening. Mechanical restriction of mouth opening is not the only clinically significant aspect, it also involves consequences for nutrition, oral hygiene, speech, and, more broadly, the overall quality of life of affected patients.

### Non-neurological causes: non-structural damage to the masticatory system

Metabolic and toxic disorders represent important non-structural, non-primary neurological causes of trismus, often mediated through muscular hyperexcitability. Metabolic disorders such as hypocalcemia may trigger muscle hyperexcitability, with trismus often presenting as an early symptom (58). Similarly, hypomagnesemia impairs neuromuscular transmission, exacerbating spastic contractions of the masticatory muscles (59). Trismus may serve as an early clinical indicator of malignant hyperthermia (60). Masseter spasm can present either in isolation or alongside hallmark systemic features, including hyperpyrexia, rhabdomyolysis, generalised rigidity, autonomic instability, and metabolic acidosis. Clinically, masseter spasm has been frequently observed following administration of succinylcholine, a depolarising neuromuscular blocking agent commonly employed for rapid-sequence intubation in emergency settings due to its rapid onset and short duration (61). The risk is particularly pronounced in paediatric patients receiving concomitant volatile anesthetics, such as halothane (62). In addition, although less common, trismus may manifest as an atypical adverse effect of etomidate, another pharmacologic agent capable of inducing muscular rigidity, and is increasingly recognised as a contributor to masseter spasm in intensive care units (ICUs) (63). The pathophysiology of drug-induced trismus likely involves dysregulation of calcium homeostasis in skeletal muscle, abnormal activation of the ryanodine receptor, and hyperactivity of muscle fibers in jaw muscles (64).

## The diagnosis algorithm of trismus

Determining the underlying etiology of restricted mouth opening remains particularly challenging due to the lack of standardized definitions and diagnostic criteria. Clinically, trismus is characterised by a limitation in mouth opening of less than 35 mm in adults, whereas the normal values range from 40 to 60 mm (65). Clinical examination is the cornerstone of diagnosis, starting from differentiating trismus from other causes of restricted mouth opening, such as mechanical obstructions (1). In this context, bedside assessments, including passive jaw movement tests, are essential for accurate evaluation. Once trismus is confirmed, identifying its underlying etiology becomes critical. A comprehensive clinical history should be obtained, documenting the onset, duration, and progression of trismus. The anamnestic inquiry should include prior trauma, infections, dental procedures, neurological manifestations, and systemic diseases. Trismus of odontogenic origin may be associated with dental pain, gingival swelling, or localised infection, whereas traumatic etiologies may present with facial or mandibular pain. Additionally, a thorough review of medication and vaccination history, particularly regarding tetanus, is recommended.

Physical examination should begin with the measurement of the interincisal distance, followed by an assessment of TMJ function and jaw range of motion. Evaluation of the orofacial soft tissues is also essential to identify signs of infection, trauma, or neoplastic lesions. The examination should be as targeted as possible, focusing on the teeth and gingiva, facial bones, TMJ, pharyngeal pillars, tonsils, uvula, and cervical region. Neurological examination should be focused on cranial nerve integrity, motor function, and the presence of generalised muscle spasms. In cases of suspected tetanus, particularly in patients with recent injuries or those from regions where tetanus is endemic, a focused physical examination to identify muscle spasms and signs of autonomic dysfunction is critical (44, 45). When infection is suspected, it is essential to obtain a complete blood count and perform culture and sensitivity testing of any purulent discharge.

Instrumental evaluation plays a critical role in identifying the underlying etiology of trismus, particularly in neurologically mediated cases. Brain imaging, including MRI and CT scan, is crucial for detecting intracranial lesions, tumours, or vascular abnormalities that may contribute to trismus (4). Nerve conduction study and trigeminal reflex study are useful tools for identifying peripheral lesions of the trigeminal system (66). Electromyography can help to identify and characterise muscle activity responsible for trismus (67). Electroencephalography is required in cases with a clinical suspicion of status epilepticus (68). Additionally, ultrasonography provides a non-invasive method for assessing masseter muscle thickness and contraction, offering real-time insights into disease progression (69). Fiber optic endoscopic evaluation of swallowing (FEES) is a valuable tool for assessing aspiration risk and airway obstruction in patients with severe trismus (70). Furthermore, quantitative jaw tracking and kinematic analysis techniques enable objective measurement of mandibular range of motion and responsiveness to treatment (71). Lastly, in cases of suspected central nervous system infection, lumbar puncture is warranted.

**Figure 1:**
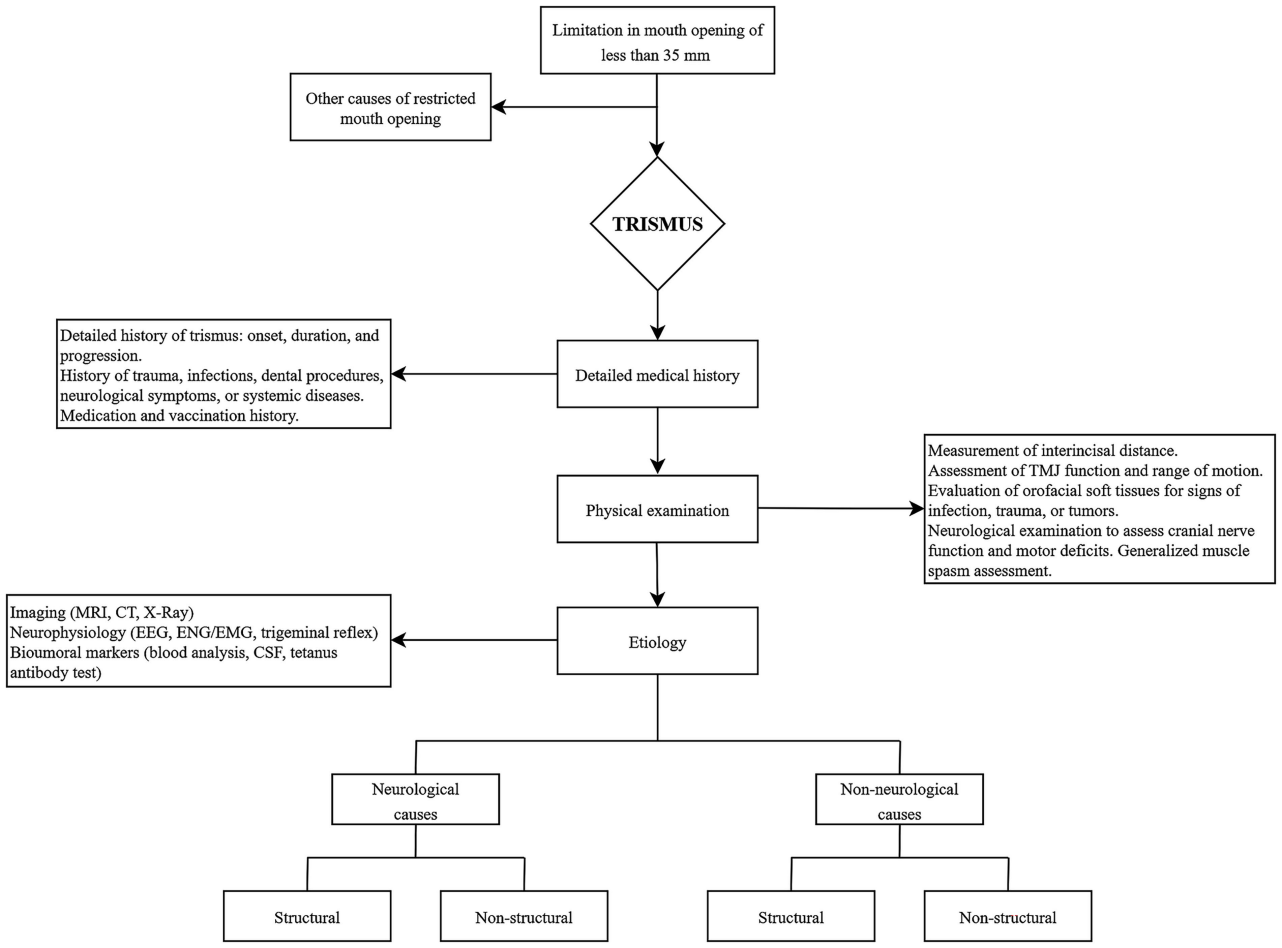
Clinical algorithm for the diagnosis of trismus.

In clinical practice, the diagnostic approach should prioritise the rapid identification of red flag conditions, including airway compromise, deep neck space infections, tetanus, central nervous system infections, and intracranial lesions. These potentially life-threatening conditions require immediate evaluation and frequently necessitate hospital-based multidisciplinary management before further etiological investigation is undertaken. The proposed diagnostic algorithm is designed as a pragmatic and clinically oriented, stepwise framework that helps clinicians differentiate neurological from non-neurological causes of trismus while simultaneously stratifying patients according to clinical urgency. In real-world settings, the algorithm supports early triage by guiding clinicians in distinguishing cases requiring emergency or inpatient management from those that can be safely investigated and treated in an outpatient setting. After exclusion of life-threatening conditions, the algorithm facilitates targeted history-taking, focused clinical examination, and the selection of appropriate instrumental investigations based on the most likely underlying etiology. See Figure 1 for a detailed diagnostic algorithm.

## Therapeutic strategies

The clinical management of trismus focuses primarily on addressing the underlying etiology. While multiple therapeutic options exist, it should be noted that most of the studies available in the literature consist of case reports or reviews, whereas data from randomized controlled trials (RCTs) are extremely limited and mainly concern rehabilitative approaches. Effective management requires a comprehensive, multidisciplinary approach that integrates pharmacological therapies, physical rehabilitation, and, in severe or refractory cases, surgical intervention (72). Symptom-directed interventions, such as non-steroidal anti-inflammatory drugs and muscle relaxants, are commonly prescribed during the acute phase and serve as the mainstay for uncomplicated, transient trismus. Discontinuation or substitution of offending agents, including neuroleptics, antiemetics, depolarising neuromuscular blockers (e.g., succinylcholine), and other known causative drugs or exogenous toxins, is crucial in the therapeutic management of trismus. In case of metabolic disturbances, prompt correction of electrolyte imbalances or other metabolic derangements contributing to muscular hyperexcitability is mandatory. As demonstrated in RCTs, Tetanus-induced trismus treatment requires the administration of tetanus immunoglobulin, alongside supportive care to control muscle spasms and respiratory distress (44, 73). Treatment of oral and maxillofacial infections causing trismus focuses on eliminating the source of infection, typically through antibiotics and, when needed, surgical drainage (74). A possible treatment for trismus, particularly in severe cases and in patients who have not responded adequately to conventional therapies, is based on local injections of botulinum toxin type A (BoNT-A) (45, 75, 76). BoNT-A acts by inhibiting acetylcholine release at the neuromuscular junction, leading to muscle relaxation and improved jaw mobility (77). It is primarily indicated for trismus of neurological origin, or for non-neurological cases in which there is no structural alteration mechanically limiting mouth opening (for example, TMJ ankylosis). Several studies, the majority being case reports, have explored the efficacy of BoNT-A injections in treating trismus, with results demonstrating significant functional improvement and symptom relief (45, 75, 76). While BoNT-A is effective for many causes of trismus, its use in tetanus-induced trismus must be considered within the broader context of tetanus management, including antitoxin administration and supportive care (76). In cases of trismus secondary to DAI, BoNT-A can help manage spasticity-related trismus, although the underlying neural damage may limit full recovery (21, 78). Baclofen, a *γ*-aminobutyric acid type B receptor agonist, is used in the management of neurological trismus due to its ability to reduce muscle spasticity, although the available evidence is limited and primarily derived from case reports (79). It exerts its effects primarily by inhibiting excitatory neurotransmitter release at the spinal and brainstem levels, leading to decreased motorneuron excitability and reduced involuntary muscle contractions (80). Baclofen is particularly effective in conditions such as spastic trismus secondary to stroke, DAI, cerebral palsy, and multiple sclerosis, where the loss of inhibitory control over the trigeminal motor nucleus results in persistent jaw closure (81). Both oral and intrathecal baclofen (ITB) administration have demonstrated efficacy in reducing trismus severity, with ITB being reserved for severe refractory cases (82). Studies indicate that early baclofen therapy can prevent complications such as TMJ dysfunction, aspiration pneumonia, and orofacial pain by promoting muscle relaxation (83). Additionally, baclofen is often combined with BoNT-A injections for synergistic effects in managing severe spasticity-related trismus (79).

Surgical treatments are reserved for refractory trismus with a confirmed structural mechanical restriction; however, the level of evidence remains limited as most available data derive from retrospective series. Coronoidectomy is the primary surgical intervention for extra-articular trismus due to temporalis muscle fibrosis or post-radiation contracture. The procedure involves the resection of the mandibular coronoid process to release the mechanical restriction caused by hyperactive or fibrotic muscles (84, 85). It typically yields an immediate and substantial increase in maximal mouth opening and has demonstrated efficacy across diverse etiologies, including neurological disorders (86, 87). However, current evidence is largely limited to small retrospective series. Long-term outcomes critically depend on early postoperative physiotherapy, because relapse due to scar contracture is common without rehabilitation. For this reason, coronoidectomy should be considered part of a combined surgical and rehabilitation treatment rather than a definitive treatment alone. Myotomy is indicated when trismus is predominantly related to muscular contracture rather than bony impingement. It may involve the masseter, temporalis, or medial pterygoid muscles, depending on the pathophysiology identified clinically and radiologically. The available evidence suggests improvement in mouth opening and pain reduction, particularly in post-radiation fibrosis and neurological spasticity; however, outcomes are less predictable compared with coronoidectomy because fibrosis may extend beyond the released muscle. Moreover, excessive muscle release may compromise masticatory efficiency, representing a relevant functional limitation (86–88). In advanced cases characterized by severe fibrosis, soft-tissue deficiency, or post-oncologic resection, release procedures alone are insufficient and reconstructive surgery becomes necessary. Free vascularized flap reconstruction involves the transplantation of vascularized tissue to restore both structural integrity and functional capacity of the affected area (89). This approach is especially indicated after head and neck cancer treatment with radiation-induced fibrosis. Although in case series, careful consideration must be given to donor-site morbidity, operative time, and patient systemic conditions must be carefully considered. Surgical intervention should be implemented according to a stepwise escalation approach and restricted to patients with clearly documented structural limitations. However, surgery cannot be considered a standalone treatment. Structured postoperative physiotherapy is indispensable to maintain the achieved mouth opening and to reduce the risk of recurrence, which remains the leading cause of long-term failure.

Lastly, physical therapy techniques, including passive stretching and jaw mobilisation exercises, are fundamental for preventing fibrosis and restoring mandibular function(1). Postoperative rehabilitation, including physical therapy and jaw exercises, is crucial to maximise the functional outcomes of surgical treatment and prevent recurrence (86, 87). Stretching exercises may be indicated after the acute phase or in patients with post-traumatic and post-operative trismus, particularly when persisting longer than 1 week. The exercises typically involve repeated attempts to open the mouth against applied resistance, usually divided into multiple daily sessions (90, 91).

## Prognosis of trismus

The prognosis of trismus is closely determined by its underlying etiology. While in many cases it is self-limiting and resolves within 2 weeks, a subset of patients experience persistent or progressive symptoms (86). For instance, trismus secondary to post-radiotherapy fibrosis often follows a protracted course and is frequently resistant to conservative management (3). This condition may lead to substantial complications, including malnutrition due to reduced oral intake, impaired airway clearance with increased aspiration risk, and poor oral hygiene predisposing to dental caries, infections, and, in severe cases, mandibular osteomyelitis. Functional impairments, particularly dysphagia and dysarthria, are common, while prolonged jaw immobility can result in temporomandibular joint degeneration from disuse atrophy (69). BoNT-A has demonstrated efficacy in mitigating fibrosis and contracture in severe cases; however, delayed diagnosis or inadequate management frequently lead to persistent dysfunction with serious nutritional and respiratory consequences (45). In tetanus, prognosis is largely dictated by infection severity and treatment timeliness, with trismus resolution representing a key marker of recovery (44). In cases associated with DAI, outcomes depend on the extent of neural damage and the success of rehabilitative interventions (21).

This review has several limitations since the narrative design did not include a quantitative evaluation of the scientific literature. Furthermore, concerning available treatments, high-level evidence is limited, and conclusions are based mainly on case reports and expert opinion or literature reviews.

## Conclusion

Trismus represents an interdisciplinary clinical challenge due to its heterogeneous etiology, including both neurological and non-neurological factors, as well as structural and non-structural causes. Understanding these diverse pathogenetic pathways is essential for comprehensive patient assessment, timely recognition of urgent conditions, and the implementation of appropriate diagnostic and therapeutic strategies across clinical and critical care settings. This review synthesises current evidence, highlighting the critical role of early diagnosis, personalised treatment strategies, and collaborative care to improve patient outcomes. Further research is needed to establish evidence-based guidelines for diagnosing and managing trismus. Additional studies on BoNT-A dosing, efficacy, and long-term benefits in the treatment of trismus are warranted. Lastly, standardising screening protocols and advancing imaging modalities could enhance early detection and therapeutic intervention, ultimately leading to better patient care.
