# Supplementary material for: An update on trismus: etiology, diagnosis and treatment
Source: Front Neurol. 2026 Mar 24;17:1758417. doi: 10.3389/fneur.2026.1758417 (PMC13053315; doi:10.3389/fneur.2026.1758417)
Supplement: Supplementary file 1 [file Data_Sheet_1.docx]

**Supplementary Material**

**Adherence to SANRA (Scale for the Assessment of Narrative Review Articles)**

This narrative review was conducted in accordance with the SANRA (Scale for the Assessment of Narrative Review Articles) recommendations to ensure methodological transparency, scientific rigor, and clarity in the synthesis of the literature. The following section describes how each SANRA item was addressed in the present manuscript.

*1. Justification of the Article’s Importance for the Readership*

The clinical and scientific relevance of trismus is outlined in the Abstract and Introduction sections. Trismus represents a complex and frequently underestimated clinical condition that can significantly impact patient quality of life, diagnostic processes, and therapeutic decision-making. The manuscript emphasises the importance of recognising trismus in neurological practice while acknowledging its multidisciplinary relevance across several medical and surgical specialties. Furthermore, the review highlights the need for improved etiological classification and structured management strategies to optimise patient care.

*2. Statement of Concrete Aims or Formulation of Questions*

The aims of the review are clearly defined in the Introduction. The manuscript aims to provide a comprehensive overview of the pathophysiological mechanisms underlying trismus, with particular emphasis on neurological aspects. Additionally, the review critically evaluates the available literature to propose a refined etiological classification distinguishing neurological and non-neurological causes, further stratified into structural and non-structural factors. Based on this classification, the manuscript proposes a structured diagnostic and therapeutic algorithm intended to support clinical decision-making in both routine and critical care settings.

*3. Description of the Literature Search*

The literature search strategy is described in detail in the Methods section under “Literature Research Strategy and Criteria.” A structured search was conducted using multiple electronic databases, including MEDLINE, PubMed, Scopus, Web of Science, EMBASE, and the Cochrane Library. The search strategy included combinations of key terms such as “trismus,” “jaw dysfunction,” and “treatment of trismus,” as well as specific therapeutic terms including “botulinum toxin type A” and “BoNT-A.” Additional search terms related to specific etiologies, including tetanus-related trismus, were also incorporated. Boolean operators were applied to refine search results.

Studies were selected based on clinical relevance and included peer-reviewed publications reporting data on the etiology, pathophysiology, diagnosis, or management of trismus. Eligible study designs included clinical studies, case reports, case series, expert reviews and RCTs. Both neurological and non-neurological causes were considered, including structural and functional mechanisms. The reference lists of selected articles were manually screened to identify additional relevant studies. As this is a narrative review, literature selection followed predefined criteria while maintaining flexibility to include seminal, highly cited, or clinically relevant publications when appropriate.

*4. Referencing*

References were selected to provide a balanced, comprehensive, and up-to-date overview of the topic. Priority was given to studies with strong clinical relevance, landmark publications, and articles addressing key aspects of trismus, including pathophysiology, classification, diagnosis, and treatment. Efforts were made to include literature from different specialties to reflect the multidisciplinary nature of trismus.

*5. Scientific Reasoning*

The review provides a critical synthesis of the available literature rather than a purely descriptive summary. Evidence from different clinical and research domains was analysed and integrated to identify areas of consensus, existing controversies, and gaps in knowledge. The proposed etiological classification of trismus was developed based on this critical appraisal, integrating current pathophysiological understanding with clinical applicability. The review also evaluates therapeutic approaches by discussing their rationale, limitations, and clinical implications.

*6. Appropriate Presentation of Data*

The manuscript presents the available evidence in a structured and clinically oriented format. Information is organised into thematic sections covering physiology, etiological classification, diagnostic approaches, and therapeutic management. Tables, figures, and a diagnostic and therapeutic algorithm are included to facilitate interpretation and practical application of the reviewed evidence. The presentation aims to ensure clarity, balance, and accessibility while maintaining scientific accuracy.
